# Survival of Misdiagnosed 2,4-Dichlorophenoxyacetic Acid Poisoning Masquerading as Organophosphorus Poisoning: A Case Report

**DOI:** 10.31729/jnma.8699

**Published:** 2024-08-31

**Authors:** Anjali Joshi, Aatish Joshi, Shubham Pant, Aakanksha Bhurtyal, Sunil Yadav

**Affiliations:** 1 Kathmandu Medical College and Teaching Hospital, Sinamangal, Kathmandu, Nepal; 2 Lumbini Medical College and Teaching Hospital, Tansen, Palpa, Nepal; 3 Seti Provincial Hospital, Dhangadhi, Kailali, Nepal; 4 Nepalgunj Medical College and Teaching Hospital, Banke, Nepal

**Keywords:** *2,4-dichlorophenoxyacetic acid poisoning*, *forced diuresis*, *herbicide*, *urinary alkalinisation*

## Abstract

Herbicide such as 2,4-Dichlorophenoxyacetic acid is commonly used in wheat growing regions and is being ingested with suicidal intent due to easy availability and lack of regulation for buying it. Various articles suggest high fatality upon ingestion of this compound. We report a rare survival of a 24-year-old male who ingested about 45 ml of the compound and presented with symptoms similar to organophosphate poisoning. Before presenting to our hospital, the patient was misdiagnosed and an atropine challenge test and gastric lavage was done. However, after presenting to our center, detailed history was taken and the bottle containing the compound was retrieved, following which the patient was shifted to the intensive care unit where urinary alkalinization and forced diuresis was done. He started getting better and was discharged on the fourth day. Detailed history taking can prevent misdiagnosis of 2,4-Dichlorophenoxyacetic acid poisoning. Early diagnosis and adequate supportive management of urinary alkalinization and forced diuresis can improve patient outcomes and reduce fatality.

## INTRODUCTION

Herbicide 2,4-Dichlorophenoxyacetic acid (2,4-D), used in wheat growing regions, belongs to the chlorphenoxy group and is rarely reported in suicidal attempts.^1^ The exposure routes include ingestion, inhalation, skin absorption, direct contact with eyes. The classical effects include gastrointestinal toxicity (corrosion of the upper gastrointestinal tract, gastrointestinal bleeding, abdominal pain, diarrhoea), musculotoxicity (muscle fibrillation, fasciculation, myalgia, rhabdomyolysis, myopathy) and neurotoxicity (seizures, ataxia, nystagmus).^2^ The presentation of the patient resembles organophosphorus poisoning, leading to misdiagnosis and unnecessary atropine therapy. We report a 24-year-old man who ingested 2,4-D, presenting with symptoms akin to organophosphorus poisoning, resulting in gastric lavage and an atropine challenge test before reaching our hospital.

## CASE REPORT

A 24-year-old male, known case of type-I diabetes mellitus, presented to the emergency department of a provincial hospital with an alleged history of ingestion of approximately 45 ml of liquid herbicide with complaints of epigastric pain, nausea, and drowsiness for one hour. Before being presented to the provincial hospital, the patient was first brought to a nearby private hospital where he was suspected as a case of organophosphorus poisoning and gastric lavage was performed. The atropine challenge test was also performed and then immediately referred to our centre for further management.

**Figure 1:**
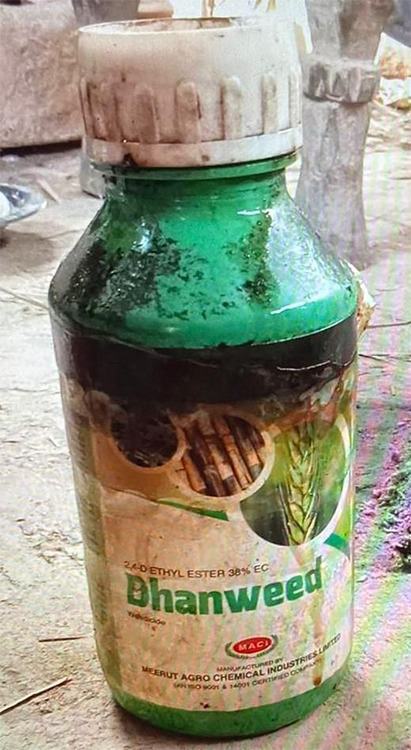
The bottle containing the herbicide which was ingested by the patient.

Upon probing further with the patient and the patient party, the patient gave a history of ingesting around three to four tablespoons of herbicide after engaging in a verbal argument with his family. The bottle containing the herbicide was retrieved and found to be 2,4-Dichlorophenoxyacetic acid, sold under the trade name Dhanweed (Figure 1). The patient party also gave history of vomiting, cough, shortness of breath, abnormal body movement, bowel incontinence, and bladder incontinence before presenting to our centre. He denied any suicidal attempts in the past.

On arrival at our centre, the patient was conscious, ill looking, and oriented to time, place, and person. His blood pressure was 100/60 mm of Hg, heart rate 121 beats per minute, respiratory rate 20 breaths per minute, and oxygen saturation was 91 percent in room air. His pupils were 3 mm in size, dilated and reactive to light. Glasgow Coma Scale was E4V4M5. On respiratory examination, a bilateral normal vesicular sound with equal air entry and no added sounds was found. A soft, non-tender abdomen with bowel sound was found on the examination of the abdomen. His laboratory values included a random blood glucose level of 408.97 mg/dl, serum urea 31.01 mg/dl, serum creatinine 0.63 mg/dl, sodium 136.3 mmol/L, potassium 4.3 mmol/L. Liver function test was also done which showed total bilirubin 0.37 mg/dl, direct bilirubin 0.17 mg/dl, aspartate aminotransferase (AST) 56.39 IU/L and alanine transaminase (ALT) 196.71 IU/L. Arterial blood gas analysis showed metabolic acidosis (pH: 7.311, pO2: 86 mm Hg, pCO2: 41.1 mm Hg, lactate: 3.6 mmol/L, HCO3: 20.3 mmol/L).

The patient was treated symptomatically. As there is no specific antidote for the treatment of 2,4-D poisoning, supportive management was carried out with intravenous crystalloids, proton pump inhibitor (pantoprazole), antibiotic (ceftriaxone 1 gram), antiemetic (ondansetron 4 milligrams) and kept nil per oral. Injection biphasic isophane insulin (30/70) was administered before and after food. Upon looking into the literature by fellow colleagues and seniors, urinary alkalinization and forced diuresis was found to have some level of benefit in the survival of the patient. The patient was then shifted to the intensive care unit where he was kept nil per oral for next 24 hours and kept on intravenous fluids, antibiotics, diuretics (furosemide), bicarbonate supplementations was done (25 mEq per hour for next 24 hour) with six hourly monitoring. Arterial blood gas analysis was performed and potassium supplementation was done with close monitoring of serum potassium six hourly. The patient improved clinically and vitals with urine output was in normal value within the first 24 hours of the intensive care unit. The patient was then given a liquid diet on the second day and arterial blood gas analysis and renal function test were repeated, both values within normal limits.

The patient was discharged on oral medications (proton pump inhibitors and antibiotics) on the fourth day with the advice to follow-up after five days. The patient was advised for an upper gastrointestinal endoscopy to rule out any erosive gastritis or stress ulcers induced by poisoning, but the patient's party denied it due to financial difficulties.

## DISCUSSION

Nepal is predominantly an agricultural country with a large percentage of the population still relying on agriculture for livelihood. This has also led to an easy and unregulated availability of herbicides, pesticides, and insecticides, which has led to increased suicidal attempts by ingesting such compounds. There is no nationwide national database to account for the total number of poisonings caused due to ingestion of such chemical compounds. However, based on research done across various health centers of Nepal, we can infer that organophosphate poison is commonly used for suicidal attempts.^3,4^ Due to lack of a registry, we were unable to find the prevalence of 2,4-D poisoning in Nepal. Upon reviewing the literature, no case was reported on 2,4-D toxicity in humans from Nepal. This could likely be due to misdiagnosis of the compound due to its similar presentation to organophosphate poisoning and lack of diagnostic methods in health centers of Nepal.

The 2,4-D is easily absorbed into the gastrointestinal and respiratory tract and rapidly eliminated unchanged in the urine, that is, the toxicokinetics of 2,4-D depends primarily on renal clearance and also plays an important role in susceptibility to 2,4-D-induced effects. The classical effects of 2,4-D include gastrointestinal toxicity (corrosion of the upper gastrointestinal tract, gastrointestinal bleeding, abdominal pain, vomiting, diarrhoea), musculotoxicity (muscle fibrillation and fasciculation, myalgia, rhabdomyolysis, myopathy), and neurotoxicity (altered mental status, seizure, ataxia, nystagmus).^2^ The production and metabolism of 2,4-D leads to the production of highly toxic compounds such as chlorophenols or dioxins that cause mitochondrial injury, dose-dependent cell membrane damage, uncoupling of oxidative phosphorylation, disruption of acetyl coenzyme, and apoptosis by free radical reaction.^5,6^ The minimum toxic dose of 2,4-D in humans in 3-4 grams and fatal dose is more than 6.5 grams in adults.^5,7^ Although the exact fatality rate was not estimated, various reports from around the world suggest that the fatal outcome is much higher upon ingestion of 2,4-D.^8-10^ Our patient had around 45 ml of the herbicide and survived which is most likely due to early diagnosis and appropriate management.

The diagnosis of 2,4-D poisoning in blood is made by liquid gas chromatography with electron-capture.^2^ However, this technology is not used commonly in Nepal. In health centres of Nepal, the diagnosis and primary management of any poisoning case is done based on the history and clinical examination of the patient, following which the investigation is sent as necessary. This increases the chance of misdiagnosis of 2,4-D poisoning as it mimics the signs and symptoms of organophosphate poisoning which happened in our case too. Only after finding the bottle was the actual poison known and the management was changed.

The 2,4-D poisoning does not have any antidote. Hence, only supportive management is done. However, the literature suggests that urinary alkalinization and forced diuresis may be beneficial to the patient. Urinary elimination works by administering intravenous sodium bicarbonate to produce alkaline urine of pH greater than 7.5 which enhances the renal excretion of the poison, thereby reducing neurotoxic and myotoxic features.^2^ Similarly, forced diuresis enhances the elimination of poison by reducing its concentration in renal tubular fluid and, therefore, the gradient for reabsorption.^2^ Durakovic et.al also recommend haemodialysis in the early phase of 2,4-D poisoning.^5^ In our case, urinary alkalinization and forced diuresis was done but haemodialysis was not done.

The 2,4-D is a potentially fatal poison. Laws and regulations should be created to ensure buying is needful. Similarly, the history and clinical examination should be prompt and detailed in order to reduce misdiagnosis so that effective management can be started as early as possible instead of administering drugs such as atropine, which is not helpful in the case of 2,4-D poisoning.

## References

[1] Bhalla A, Suri V, Sharma N, Mahi S, Singh S (2008). 2, 4-D (ethyl ester) poisoning: experience at a tertiary care centre in northern India.. Emergency medicine journal..

[2] Rajendran A, Mahalingam S, Babu GR, Rajendra KR, Nathan B (2021). 2, 4-Dichlorophenoxyacetic Acid Poisoning Mimicking as Organophosphorus Poisoning.. Cureus..

[3] Gyenwali D, Vaidya A, Tiwari S, Khatiwada P, Lamsal DR, Giri S (2017). Pesticide poisoning in Chitwan, Nepal: a descriptive epidemiological study.. BMC public health..

[4] Paudyal BP (2005). Poisoning: pattern and profile of admitted cases in a hospital in central Nepal.. JNMA J Nepal Med Assoc..

[5] Durakovic Z, Durakovic A, Durakovic S, Ivanovic D (1992). Poisoning with 2, 4-dichlorophenoxyacetic acid treated by hemodialysis.. Archives of toxicology..

[6] Bukowska B (2003). Effects of 2, 4-D and its metabolite 2, 4-dichlorophenol on antioxidant enzymes and level of glutathione in human erythrocytes.. Comparative Biochemistry and Physiology Part C: Toxicology & Pharmacology..

[7] Clarke EG (1969). Isolation and identification of drugs..

[8] Jearth V, Negi R, Chauhan V, Sharma K (2015). A rare survival after 2, 4-D (ethyl ester) poisoning: Role of forced alkaline diuresis.. Indian Journal of Critical Care Medicine..

[9] Keller T, Skopp G, Wu M, Aderjan R (1994). Fatal overdose of 2, 4-dichlorophenoxyacetic acid (2, 4-D).. Forensic science international..

[10] Jorens PG, Heytens L, De Paep RJ, Bossaert L, Selala MI, Schepens PJ (1995). A 2, 4-dichlorophenoxyacetic acid induced fatality.. European Journal of Emergency Medicine..

